# Combined use of EpCAM and FRα enables the high-efficiency capture of circulating tumor cells in non-small cell lung cancer

**DOI:** 10.1038/s41598-018-19391-1

**Published:** 2018-01-19

**Authors:** Luojun Chen, Min Peng, Na Li, Qibin Song, Yi Yao, Bin Xu, Huali Liu, Peng Ruan

**Affiliations:** Department of Oncology, Renmin Hospital of Wuhan University, Wuhan, 430060 China

## Abstract

Circulating tumor cells (CTCs) provide a new approach for auxiliary diagnosis, therapeutic effect evaluation, and prognosis prediction for cancer patients. The epithelial cell adhesion molecule (EpCAM)-based separation method (CellSearch) showed good clinical use in multiple types of cancer. Nevertheless, some non-small cell lung cancer (NSCLC) tumor cells have a lower expression of EpCAM and are less frequently detected by CellSearch. Here, we present a highly sensitive immunomagnetic separation method to capture CTCs based on two cell surface markers for NSCLC, EpCAM and Folate receptor alpha (FRα). Our method has been demonstrated to be more efficient in capturing NSCLC cells (P < 0.01) by enriching three types of CTCs: EpCAM^+^/FRα^−/low^, EpCAM^−/low^/FRα^+^, and EPCAM^+^/FRα^+^. In 41 NSCLC patients, a significantly higher CTC capture rate (48.78% vs. 73.17%) was obtained, and by using a cutoff value of 0 CTC per 2 ml of blood, the sensitivities were 53.66% and 75.61% and the specificities were 100% and 90% for anti-EpCAM-MNs or a combination of anti-EpCAM-MNs and anti-FRα-MNs, respectively. Compared with the tumor-specific LT-PCR based on FRα, our method can isolate intact FRα^+^ CTCs, and it is advantageous for additional CTC-related downstream analysis. Our results provide a new method to increase the CTC capture efficiency of NSCLC.

## Introduction

Circulating tumor cells (CTCs) are cancerous cells shed in the bloodstream that eventually lead to distant metastases^1,2^. Many studies have demonstrated that CTCs can be a biomarker in auxiliary diagnosis^3–5^, therapeutic effect evaluation^6^, gene mutation analysis^7^, recurrent metastasis monitoring^8,9^, and prognosis prediction^10–13^ for cancer patients. However, CTCs are extremely rare, occurring at frequencies as low as 1 CTC per 10^6^–10^7^ leukocytes^14^, which requires that the detection method must have high sensitivity and specificity. Recently, different detection methods have emerged, such as immunology-based methods^15^, microfluidics devices^16,17^, filter-based methods^1^, aptamer-based technologies^18,19^, hierarchical assembled ITO nanowire array^20^, ligand-targeted PCR (LT-PCR)^21^, but few CTC detection methods have been approved for routine clinical use. The only one that has been approved by the US FDA is CellSearch system (Veridex, Raritan, NJ), which is an immunology-based platform that uses the epithelial cell adhesion molecule (EpCAM) as the capture target^15^. It has shown good clinical use in multiple types of advanced cancers, including breast cancer, prostate cancer, and colon cancer; however, clinical studies showed low sensitivity of the EpCAM-based enrichment in the CTC detection of NSCLC patients^22^. This was mainly due to the epithelial to mesenchymal transition (EMT) during metastasis, with the loss of more epithelium-like CTCs^23^. Thus, the selection of tumor-specific antigens on the cell surface is the key to improving the CTC detection rate.

Folate receptor alpha (FRα), which is a glycosylated phosphatidylinositol-anchored glycoprotein, is highly expressed in a variety of cancers, including head and neck cancer^24^, breast cancer^25^, and ovarian cancer^26^, as well as NSCLC^27–30^. Studies have shown that 72–83% of patients with lung adenocarcinoma overexpress FRα on the cell membrane, but there is limited expression in normal adult tissues^27,29^. Furthermore, FRα expression appears to be associated with patients who have never smoked^29^, the EGFR gene mutation^27,30^, p53 wild-type^30^, low histologic grade, well-differentiated^29,30^, better responses to antifolate chemotherapy^27^ and a favorable prognosis^30^. Indeed, FRα has been used as a therapeutic target in clinical trials in NSCLC and ovarian cancer^31–34^. Now, ligand-targeted PCR (LT-PCR), using folate-crosslinking nucleotide fragments as a detection probe, demonstrated good sensitivity (74.4%) and specificity (86.6%)^35^, but LT-PCR can only obtain the number of CTCs; it cannot analyze the molecular pathogenesis, such as mutation detection. An intact CTCs that be captured and fluorescently labeled by immunomagnetic nanospheres can be visualized and isolated single CTC by the semiautomatic DEPArray system (Silicon Biosystems, Italy) and subsequent gene expression-level or mutation can be analyzed at the single CTC level by using whole genome amplification (WGA) analysis or next-generation sequencing (NGS). Therefore, FRα is an ideal immune capture target for CTC detection.

Combining different immune capture targets helps improve the CTC detection rate^36–39^. A study found that FRα-positive (FRα^+^) CTC levels were significantly higher in EpCAM-negative (EpCAM^−^) fractions than in EpCAM-positive (EpCAM^+^) fractions in NSCLC patients^21^; this demonstrates that the expression of EpCAM and FRα in NSCLC were heterogeneous. Based on this heterogeneous expression pattern, the combination of FRα and EpCAM as the targets of immunomagnetic sorting technology can improve the sorting rate by enriching three types of CTCs: EpCAM^+^/FRα^−/low^, EpCAM^−/low^/FRα^+^, and EPCAM^+^/FRα^+^. In this study, we demonstrated the combined use of EpCAM and FRα as capture targets in NSCLC cell lines and NSCLC patients with higher efficacy and sensitivity, suggesting their translational potential for future development of CTC detection methods.

## Results

### Validation of CTC-capture antigens (EpCAM and FRα) and CTC-identification antigens (CK and CD45)

**Figure 1:**
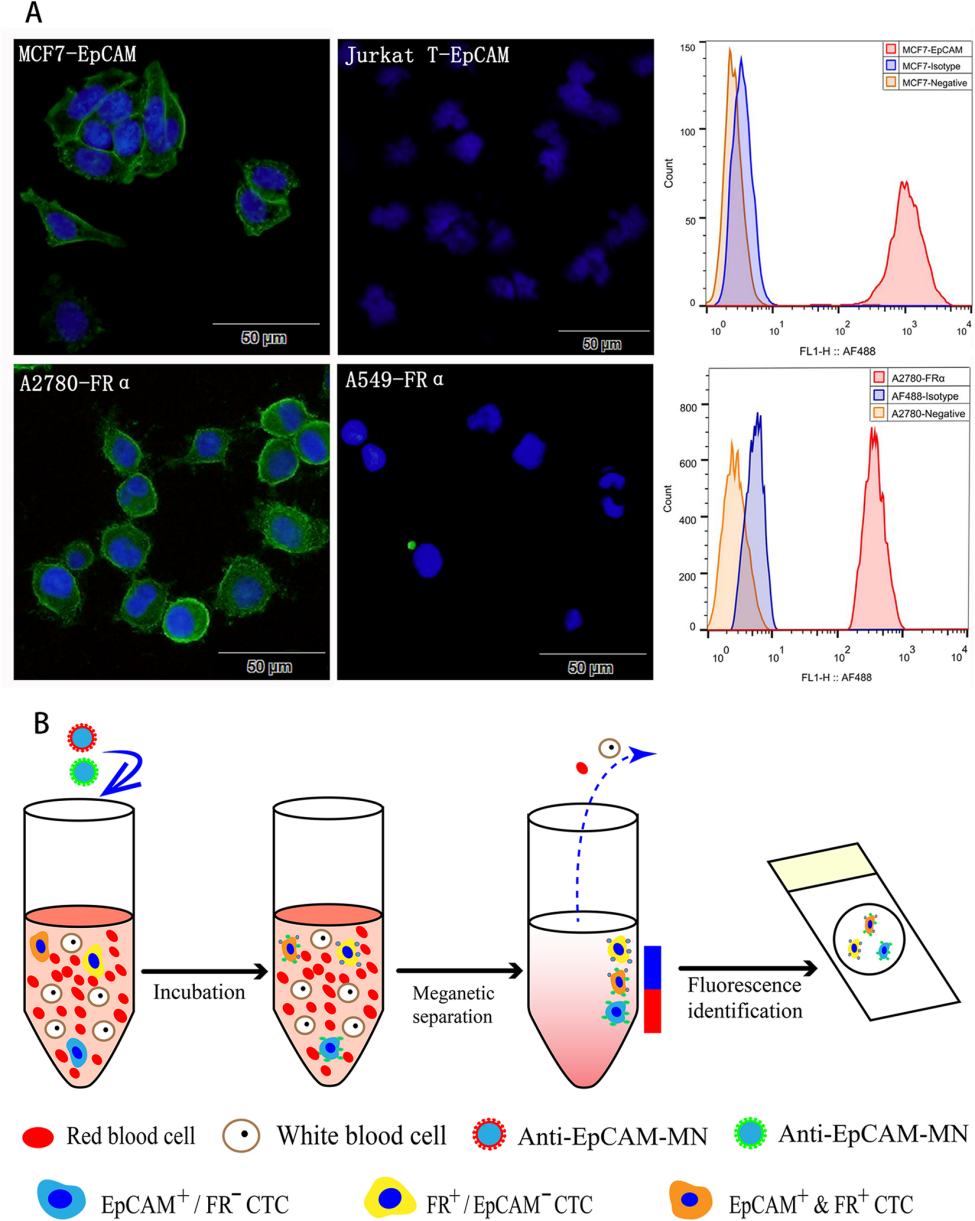
(**A**) Immunofluorescent (left) and flow cytometry (right) detection of the EpCAM expression in MCF7 cells and the FRα expression in A2780 cells. EpCAM and FRα stained with Alexa Fluor^®^ 488 are green at an excitation of 488 nm, and the nuclei stained with DAPI are blue at an excitation of 405 nm. As a negative control, we used EpCAM to stain Jurkat cells, which are EpCAM negatively expressed, and we used FRα to stain A549 cells, which are FRα negatively expressed. Histograms of flow cytometric analysis: MCF7 cells (top) were stained with anti-EpCAM antibodies (red) and A2780 cells (bottom) were stained with anti-FRα antibodies (red), the negative control was autofluorescent (orange), and the isotype was mouse IgG plus secondary antibody (blue). (**B**) Schematic of our CTCs enrichment strategy. Anti-EpCAM-MNs and Anti-FRα-MNs was added to the whole blood and after incubation, magnetic separation and fluorescence identification, the cells of DAPI^+^/CK^+^/CD45^−^ were defined as CTC.

First, we detected the feasibility of the anti-EpCAM and anti-FRα antibodies using two methods: immunofluorescence (IF) and flow cytometry. Flow cytometry showed that the anti-EpCAM antibody could obtain 97.47% of EpCAM highly expressing MCF7 cells, while the anti-FRα antibody could obtain 99.92% of FRα highly expressing A2780 cells. The immunofluorescence demonstrated that the anti-EpCAM antibody could combine with MCF7 cells but not Jurkat cells (EpCAM^-^), and the anti-FRα antibody could combine with A2780 cells but not A549 cells (FRα^−^). EpCAM and FRα were expressed on the cell membrane (Fig. 1(A)), so these antibodies that capture target cells have good sensitivity and specificity. We then used immunofluorescence to detect the expression of EpCAM and FRα in the NSCLC cell lines. As shown in Fig. S1, the expression of the EpCAM antigen was very low or negative in the NSCLC cells, while the FRα antigen in SPC-A-1 and H157 cells was high, but very low or negative in H1299, H460, and A549 cells. Furthermore, we used immunofluorescence to detect the expression of CD45, CK19, EpCAM, and FRα antigens in healthy donors’ peripheral blood. As shown in Fig. S2, no CK19, EpCAM, and FRα antigens were expressed in the peripheral blood mononuclear cells (PBMCs), and CD45 was highly expressed in the PBMCs. Therefore, the antigens of EpCAM and FRα specifically captured CTCs but not white blood cells, CK19 specifically identified CTCs, and CD45 was a sensitive antigen to eliminate white blood cells.

### Optimization of IMN-based cell sorting strategy and feasibility detection

**Figure 2:**
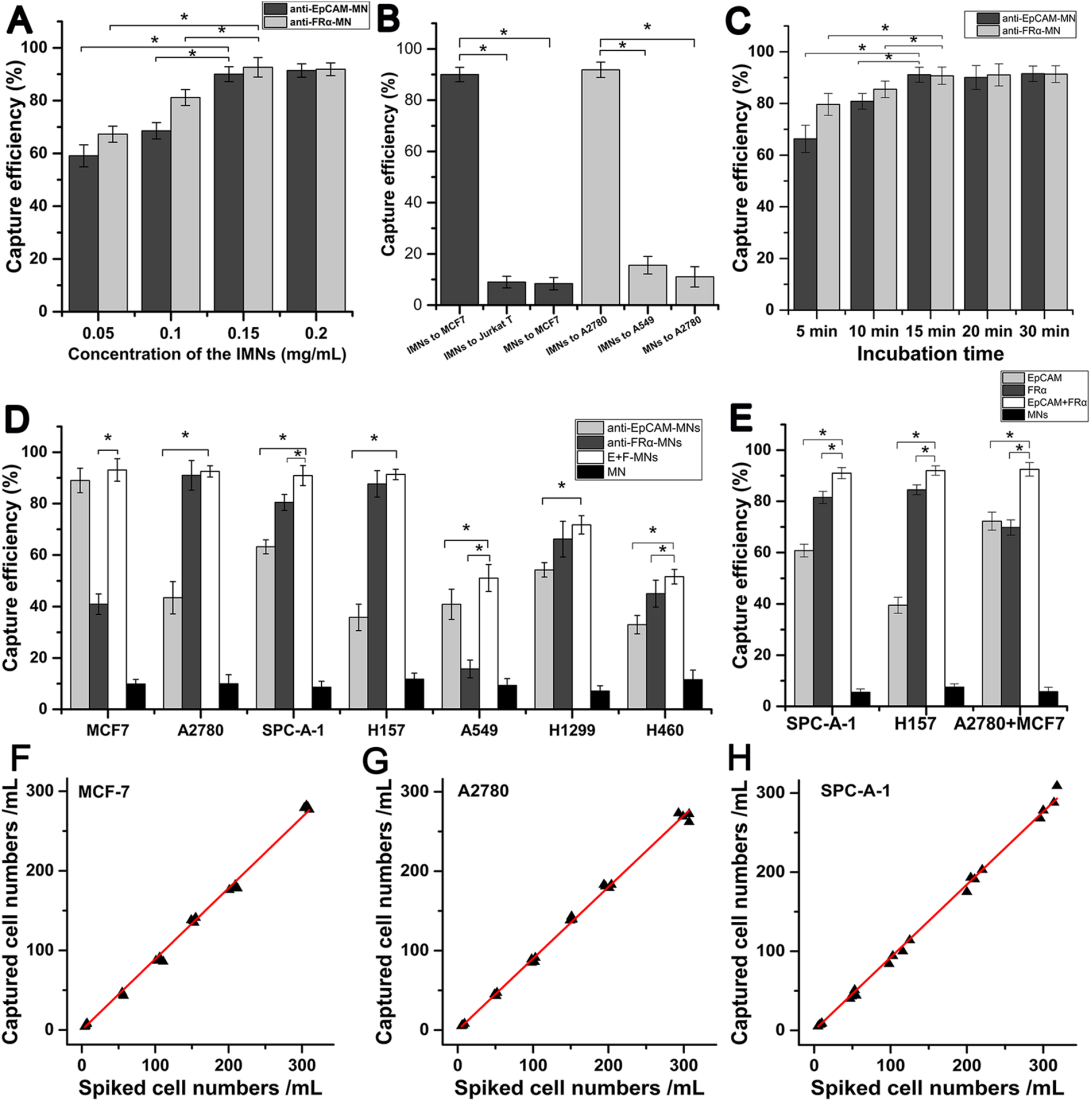
Efficiencies of IMNs to capture target cells. (**A**) The capture efficiencies at different concentrations of anti-EpCAM-MNs to capture MCF7 cells (black) and anti-FRα-MNs to capture A2780 cells (gray). (**B**) Specificity detection of IMNs. Black column shows capture efficiencies of anti-EpCAM-MNs to MCF7 and Jurkat cells and MNs to MCF7 cells. The gray column shows the capture efficiencies of anti-FRα-MNs to A2780 and A549 cells and MNs to A2780 cells. (**C**) The capture efficiencies at different incubation times of anti-EpCAM-MNs to capture MCF7 cells (black) and anti-FRα-MNs to capture A2780 cells (gray). (**D**) The capture efficiencies of anti-EpCAM-MNs (light gray) or anti-FRα-MNs (dark gray) used alone or in combination (white) to capture MCF7, A2780, and five types of NSCLC cells; the black column shows the capture efficiencies of unmodified MNs to cells. (**E**) Capture efficiencies in mimicking clinical samples of anti-EpCAM-MNs (light gray), anti-FRα-MNs (dark gray), combined anti-EpCAM-MNs and anti-FRα-MNs (white), and unmodified MNs (black) to capture SPC-A-1, H157, or a mixture of A2780 and MCF7 cells. (**F**) The regression analyses plots of recovered vs the number of spiked MCF7 cells detected by anti-EpCAM-MNs. (**G**) The regression analyses plots of recovered vs the number of spiked A2780 cells detected by anti- FRα-MNs. (**F**) The regression analyses plots of recovered vs the number of spiked SPC-A-1 cells detected by the combination of anti-EpCAM-MNs and anti-FRα-MNs.

To optimize the working conditions of the CTC detection methods, we tested the capture efficiency at different concentrations of immunomagnetic nanospheres (IMNs) and at different assay times. With the increase of the IMN concentration, the capture efficiency increased until the concentration reached 0.15 mg/ml (Fig. 2(A)); at this concentration, more than 90.0% of MCF7 can be captured by anti-EpCAM-MNs and 92.6% of A2780 can be captured by anti-FRα-MNs. At this concentration, IMNs can barely capture Jurkat cells (anti-EpCAM-MNs, <8.9%) and A549 cells (anti-FRα-MNs, <15.6%), and unmodified MNs could hardly capture MCF7 (<8.4%) and A2780 cells (<11.0%), indicating that the binding between the IMNs and cells was effective and specific (Fig. 2(B)). In addition, 15 min of incubation enabled IMNs to capture more than 90.0% of MCF7 cells (anti-EpCAM-MNs) and A2780 cells (anti-FRα-MNs) in whole blood. Thus, 15 min of incubation was sufficient for IMNs to bind the target cells (Fig. 2(C)). Then, we tested the capability of IMNs to capture rare target cells in synthetic CTC samples, which were prepared by spiking stained target cells into whole blood with concentrations of 5–300 cells/ml. The relationship between the number of recovered vs. the number of spiked tumor cells was linear, and regression analysis obtained y_EpCAM_ = 0.8913 × (R² = 0.99, 95% CI = 0.8772–0.9054) (Fig. 2(F)) and y_FRα_ = 0.9005 × (R² = 0.99, 95% CI = 0.8882–0.9129) (Fig. 2(G)). From all of these experimental results, it can be concluded that IMNs were able to capture rare target tumor cells from whole blood efficiently, specifically, and quickly.

### Combination of anti-EpCAM-MNs and anti-FRα-MNs for NSCLC cell line enrichment

The CTC-enrichment efficiency of anti-EpCAM-MNs alone or a combination of anti-EpCAM-MNs and anti-FRα-MNs was compared using five types of NSCLC cells lines (1.0 × 10^5^ cells/mL in PBS) that expressed different levels of EpCAM and FRα. MCF7 and A2780 cells were the respective positive controls, and unmodified MNs were used to treat cells to investigate the specificity. Figure 2(D) shows the detection rate using a combination of anti-EpCAM-MNs and anti-FRα-MNs to enrich recovered cells from spiked cells. Compared with the anti-EpCAM-MN enrichment approach, the combination of anti-EpCAM-MNs and anti-FRα-MNs in parallel allowed the capture of five types of NSCLC cells with higher efficiency (P < 0.01). The result demonstrates that the method of using a combination of antibodies (anti-EpCAM and anti-FRα) can improve the enrichment efficiency of NSCLC cells.

Then, we compared the enrichment efficiency using rare tumor cells to mimic clinical samples (rare FRα highly expressing SPC-A-1 and H157 NSCLC cells were spiked into healthy donors’ blood). In order to prove that the high enrichment efficiency occurred via the capture of EpCAM-positive cells and FRα-positive cells, we used a mixture of A2780 (FRα^+^/EpCAM^−/low^) and MCF7 (EpCAM^+^/FR^−/low^) cells as the control. Compared with the use of IMNs alone, the capture rate could reach 90% with the combination of anti-EpCAM-MNs and anti-FRα-MNs, which is about 10–50% higher than anti-EpCAM-MNs alone (P < 0.01) in mimics of NSCLC clinical samples. In the mixture of A2780 and MCF7 cells samples, the capture rate was 92% using a combination of anti-EpCAM-MNs and anti-FRα-MNs, which was about 20% higher than that of anti-EpCAM-MNs or anti-FRα-MNs alone (P < 0.01) (Fig. 2(E)). Therefore, we demonstrated that the higher enrichment efficiency occurred via the capture of two antigens (EpCAM and FRα) on the cell membrane.

Furthermore, we analyzed the relationship between the CTC capture rate and the CTC levels by spiking rare SPC-A-1 cells into the whole blood at concentrations of about 5–300 cells/ml. The relationship between the number of recovered vs. the number of spiked tumor cells was linear, and regression analysis obtained y = 0.9216 × (R² = 0.99, 95% CI = 0.9072–0.9359) (Fig. 2(H)).

### CTC detection in NSCLC patients and FRα+ CTCs subtype analyses

**Figure 3:**
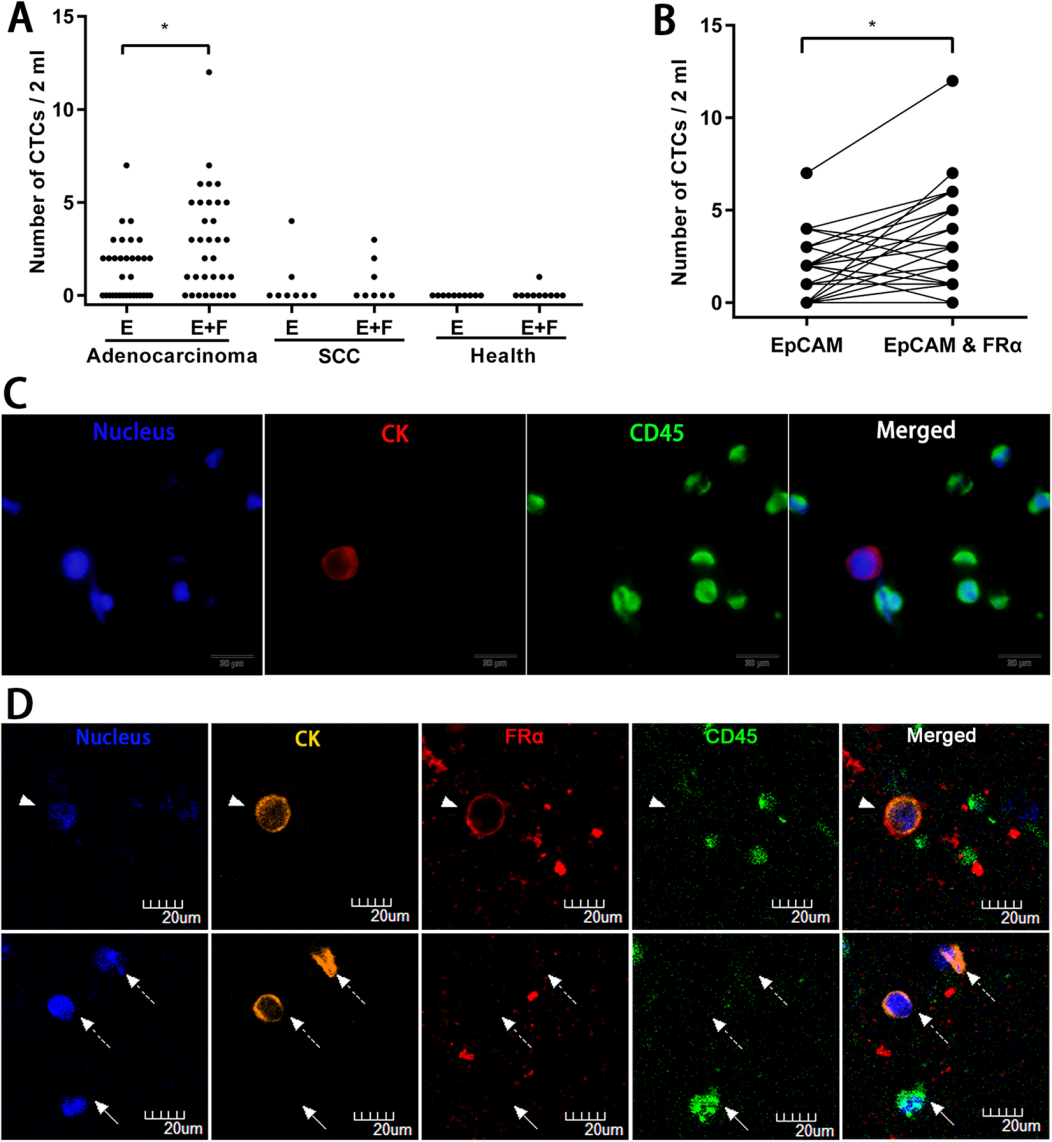
(**A**) CTC levels of 33 adenocarcinoma patients, 8 squamous cell carcinoma patients, and 10 healthy donors identified by anti-EpCAM-MNs or a combination of anti-EpCAM-MNs and anti-FRα-MNs. (**B**) Paired comparison shows the number of CTCs detected by anti-EpCAM-MNs or a combination of anti-EpCAM-MNs and anti-FRα-MNs. (**C**) Fluorescence microscopic images of cells isolated from NSCLC patients and identified with the three-color Immunofluorescent staining. The cells were stained with Alexa Fluor^®^ 568-labeled anti-CK19 monoclonal antibody (red), Alexa Fluor^®^ 488-labeled anti-CD45 monoclonal antibody (green), and DAPI (blue). The cells with CK19-positive (red) and DAPI-positive (blue) but CD45-negative (green) phenotypes were enumerated as CTCs. (**D**) Confocal fluorescence microscopic images of cells isolated from one NSCLC patient that were identified with the three-color ICC. The cells were stained with Alexa Fluor^®^ 568-labeled anti-CK19 (orange), Alexa Fluor^®^ 647-labeled anti-FRα (red), and Alexa Fluor^®^ 488-labeled anti-CD45 (green) and DAPI (blue). The captured cells contain CK^+^/FRα^+^/CD45^−^ and CK^+^/FRα^−^/CD45^−^ features.

The blood samples taken from 41 NSCLC patients and 10 healthy donors were tested with anti-EpCAM-MNs or a combination of anti-EpCAM-MNs and anti-FRα-MNs. We detected CTCs using two methods in a blind comparison study. The CTC levels of NSCLC patients and healthy donors identified by each method is plotted in Fig. 3(A) and the results from identical NSCLC patients are shown in Fig. 3(B). The results show that the detection rate was 48.8% (20/41) using anti-EpCAM-MNs but 73.2% (30/41) using a combination of anti-EpCAM-MNs and anti-FRα-MNs, and a 24.39% higher CTC detection rate was achieved with a combination of EpCAM and FRα (p < 0.001) in the NSCLC patients. In one NSCLC patient, 7 CTCs/2 mL were detected in one tube of blood using anti-EpCAM-MNs, and 12 CTCs/2 mL were detected in another tube of the same patient’s blood using the combination of anti-EpCAM-MNs and anti-FRα-MNs. The area under the receiver operating characteristic curve (AUC-ROC) analysis (Fig.S3) showed that the combination of anti-EpCAM-MNs and anti-FRα-MNs had a higher AUC (0.8585, 95% CI: 0.7579–0.9592, p = 0.0005) than that of anti-EpCAM-MNs alone (0.7683, 95% CI: 0.6384–0.8982, p = 0.0091). By using the cutoff value of 0 CTC per 2 ml of blood, the sensitivities were 53.66% and 75.61%, and the specificities were 100% and 90%, for anti-EpCAM-MNs or a combination of anti-EpCAM-MNs and anti-FRα-MNs, respectively. In healthy donors, 0 CTCs/2 mL were detected in 9 subjects, while “false positive” CTCs were detected in 1 subject according to the CTC definition of DAPI^+^/CK^+^/CD45^−^ cells (Fig. 3(A)). We think that this may have been caused by the contamination of normal vascular endothelial cells during the process of venepuncture, which also reflects the current definition (DAPI^+^/CK^+^/CD45^−^) of CTC criteria for the existence of certain defects.

Furthermore, we analyzed the relationship between the capture rate and the histological type. In adenocarcinoma patients, the detection rate was 54.55% (18/33) and 75.75% (25/33) for anti-EpCAM-MNs or a combination of anti-EpCAM-MNs and anti-FRα-MNs, and a significant improvement in enrichment efficiency was obtained with a combination of anti-EpCAM-MNs and anti-FRα-MNs (p = 0.001). However, in squamous cell carcinoma, the capture rate was 25% (2/8) and 37.5% (3/8) for these two methods, so no statistically significant difference was observed (p > 0.05) (Fig. 3(A)). These findings are consistent with previous reports describing positive FRα expression in the majority of adenocarcinomas but in a minority of squamous cell carcinomas^27,29,30^. An image gallery of representative CTCs from NSCLC is shown in Fig. 3(C). Furthermore, we evaluated the FRα expression in CTCs isolated by our CTC detection methods from one clinical NSCLC patient. Four-color immunofluorescence staining was used to characterize the different cell types that were isolated from one patient’s whole blood samples (Fig. 3(D)). The captured cells contained CK^+^/FRα^+^/CD45^−^ and CK^+^/FRα^−^/CD45^−^ features.

## Discussion

Tumor heterogeneity is a hallmark of cancer^40^, and analyses of CTCs offer a more rational approach to discern tumor heterogeneity compared with single tissue biopsy^41^. CTCs that arise from multiple sites theoretically contain all tumor-derived information. However, due to the extreme rarity, high heterogeneity and the limitations in detection technology, CTC analysis is complementary to tissue biopsy, depending upon the context of the application. The key clinical application include the auxiliary diagnosis^3–5^, therapeutic effect evaluation^6^, gene mutation analysis^7^, recurrent metastasis monitoring^8,9^, and prognosis prediction^10–13^.

In this study, we synthesized two immunomagnetic nanospheres that were modified with anti-FRα and anti-EpCAM antibodies, respectively, and applied them to CTC assays of NSCLC cell lines; the assay for the FRα^+^ cells was demonstrated to be both sufficiently sensitive and specific to detect FRα highly expressing NSCLC cell lines of SPC-A-1 and H157 cells. Then, we assayed it in NSCLC patients’ blood samples. In the blind comparison study of our method compared with the anti-EpCAM-MNs, we obtained a significantly higher CTC-positive rate (73.17% vs. 48.78%), and it was also higher compared with what had been reported for the CellSearch platform (23–42% of stage IV NSCLC patients had CTC counts ≥ 1)^42^. AUC-ROC analysis showed the high value of our method compared with anti-EpCAM-MNs. By using the cutoff value of 0 CTC per 2 ml of blood, the sensitivities were 75.61% and the specificities were 90% for our method, which were the same as those for LT-PCR based on the FRα CTC detection method^28^. Comparable to the CTC detection method of combining anti-EpCAMs and anti-cytokeratins, which require the breaking open of the cells^38,39^, our method can isolate more intact CTCs; it may be beneficial for CTC-related studies, such as mutation detection, and further CTC-related downstream analyses.

Concerning histopathologic type, we observed a significant difference between the two capture methods in adenocarcinoma (p = 0.001), but there was no statistically significant difference in squamous cell carcinoma. These findings are consistent with previous reports describing positive FRα expression in the majority of NSCLC patients but its absence in squamous cell carcinoma^27,29,30^.

The clinical value of FRα has been explored in several cancers in recent years. A study reported that the patients with high expression of FRα-positive CTCs appear to have superior responses to pemetrexed than those with low expression in NSCLC^43^. Another study demonstrated that patients with higher FRα expression had better prognoses than those with lower FRα expression in ovarian cancer^30^. Farletuzumab, which is a humanized monoclonal antibody that binds to FRα, is currently being investigated in phase II clinical trial (clinical Trials.gov identifier: NCT01218516) in NSCLC adenocarcinoma patients. More experiments need to be done for the subsequent analysis of FRα^+^ CTCs. In our study, the number of tested samples and the follow-up time are ongoing to assess the correlation between FRα^+^ CTCs and clinical outcome.

Efficient capture method is the basis of CTCs detection, but getting the number of CTCs does not show all the role of CTCs detection in the individual and precise medical. The nondestructive release of CTCs and further *in vitro* culture is an important process for genotyping and drug screening. However, there are challenges to effectively capture and nondestructive release CTCs in peripheral blood. To solve these problems, researchers have undertaken numerous efforts to ameliorate CTC detection technology. CTC detection technology consists of two steps, capture and identification. The scarce availability of “gripper” that can be used for capture is a major hindrance to CTC detection. At present, optional CTC capture grippers include aptamers and antibodies. Aptamers are single-stranded DNA or RNA oligonucleotides with affinities and specificities for their target, and have some anticipated advantages comparable to those of antibody/antigen interactions. Aptamers can release the bound target without harsh treatment, for example, the introduction of a sequence complementary to compete off the binding of the target^44,45^, or be removed with thermal denaturation^46^ or wash steps using endonucleases^47^. Wang et.al report the viability of the released cells was nearly 99% using complementary nucleic acid sequences to release binding cells^44^. For antibody/antigen interaction, more harsh treatment will need to release the bound target, such as enzyme degradation^48^. At present, a lot of preclinical research used the aptamer-based method to detect cancer cells in buffer solution and can get high detection rate, but a few of them reported the detection rate in whole blood solution. Herr’s group reported the capture rated can get to 80% when using aptamers (sgc8)-magnetic particle to detect cancer cells in buffer solution but the recovery efficiency of target cells from whole blood sample was lower at 40%^49^. The reason may be that aptamers can’t completely switch into well-defined secondary structures in blood with complex composition. This may lead to nonspecifically captured and insufficient interaction between particle-loaded aptamers and targeted cells in whole blood. Though aptamer-based technologies hold great potential, most of the aptamer-based platforms remain in laboratory settings and lack of further clinical application. Thus, more work needs to be done to improve capture efficiency in the complex milieu. For identification, immunostaining has been utilized for positive CTC identification in the CellSearch system. Apart from specific antigen expression, the level of gene transcription and chromosome abnormalities were also used for CTC identification. However, these identification methods inevitably damaged cell vitality. More work needs to be done that does not affect the cell vitality in the identification process.

## Conclusion

This study demonstrated a new approach using a combination of EpCAM and FRα as CTC-capture targets to increase the sensitivity of CTC detection in NSCLC efficiently, specifically, and quickly. With this strategy, more heterogeneous CTCs, including EpCAM^+^/FRα^−/low^, EpCAM^−/low^/FRα^+^, and EPCAM^+^/FRα^+^ tumor cells, can be captured. Because the separation process does not require penetration of the cell membrane, the captured CTCs are intact and viable, and they are conducive to subsequent analysis, such as single-cell sequencing. Despite this promising result, further studies need to be done to assess the clinical validity of this method in NSCLC patients.

## Methods

### Cell culture

Human NSCLC A549, H1299, H157, H460, SPC-A-1 cell lines, human breast cancer MCF7 cell line, human ovarian cancer A2780 cell line, human T cell leukemia Jurkat cells were obtained from China Center for Type Culture Collection. MCF7 cells were cultured in DMEM (Life Technologies, Carlsbad, USA) with 10% FBS (ScienCell, Carlsbad, CA, USA) and others were cultured in RPMI 1640 (Life Technologies, Carlsbad, USA) with 10% FBS at 37 °C in a humidified atmosphere with 5% CO2.

### Immunofluorescent staining and flow cytometry

For immunofluorescence, SPC-A-1, H157, H460, H1299, A549, A2780 cells were fixed in 4% paraformaldehyde (Servicebio, Wuhan, China) for 10 min, blocked with 2% BSA (Servicebio, Wuhan, China) for 30 min, and then processed with anti-EpCAM (SAB4700423, Sigma-Aldrich, St. Louis, USA) and anti-FRα (MAB5646, R&D Systems, Minneapolis, USA) monoclonal antibodies overnight at 4 °C. After thoroughly washing with PBST (3 × 5 min), the cells were then incubated with Alexa Fluor 488 conjugated donkey anti-mouse IgG (H + L) secondary antibody (A21202, Life Technologies, Carlsbad, USA). Negative control slides were incubated with irrelevant mouse IgG primary and secondary antibodies. Nuclear staining was visualized using DAPI (Servicebio, Wuhan, China). After thoroughly washing with PBST, cells were coverslipped using antifade mounting medium (Servicebio, Wuhan, China). As a negative control, the primary antibody was omitted, and all of the other steps were similar to those described. Fluorescence images were obtained by Olympus fluorescence microscope BX63 (Olympus, Japan).

For flow cytometry, MCF7 and A2780 cells were split into single cells with 0.25% Trypsin-EDTA (Life Technologies, Carlsbad, USA), then fixed with 4% paraformaldehyde, blocked with 2% BSA, and incubated with anti-EpCAM and anti-FRα monoclonal antibodies for 30 min at 37 °C. After washing with PBST, the cells were incubated with Alexa Fluor 488 conjugated donkey anti-mouse IgG (H + L) secondary antibody, isotype controls were incubated with irrelevant mouse IgG primary and secondary antibodies, and the negative control received nothing. The fluorescence signal was detected by three-laser FACSCalibur (BD Biosciences, San Jose, CA) and analyzed using FlowJo software, version X10.0 (TreeStar Inc.).

### Detection of the expression of CD45, CK19, EpCAM, and CK antigens in peripheral blood mononuclear cells

We transferred 4 ml of healthy donors’ whole blood to a 15-ml Falcon tube (Corning, Lowell, MA, USA) and gently mixed it with red blood cell lysis buffer (Servicebio, Wuhan, China). After approximately 5 min (when the color of the blood changed to a transparent cherry red), cells were immediately centrifuged at 400 g for 10 min at 4 °C. They were resuspended in PBS and coated on polylysine-coated slips, then fixed with 4% paraformaldehyde (10 min), permeabilized with 0.1% Triton X-100 (10 min), blocked with 2% BSA (30 min), and stained with 30 μg/mL of DAPI, Alexa Fluor^®^488 conjugated anti-CD45 antibody (ab197730, Abcam, Cambridge, UK), and Alexa Fluor^®^568 conjugated anti-Cytokeratin 19 antibody (ab203445, Abcam, Cambridge, UK). After thoroughly washing with PBST, the cells were coverslipped using antifade mounting medium. As a negative control, the primary antibody was omitted, and all of the other steps were similar to those described. Fluorescence images were obtained by Olympus fluorescence microscope BX63 (Olympus, Japan).

### Construction of antibody-modified magnetic nanospheres

We used carbodiimide chemistry to cross-link the antibody with the Sera-Mag^®^ SpeedBeads™ magnetic nanospheres (1 mg/mL, Sigma-Aldrich, St. Louis, USA) were activated by N-(3-dimethylaminopropyl)-N′-ethylcarbodiimide (EDC) (Sigma-Aldrich, St. Louis, USA) and N-hydroxysuccinimide (NHS) (Sigma-Aldrich, St. Louis, USA) and then reacted with the antibody for about 4 h with continuous shaking at room temperature, then finally stored with PBS (containing 0.05% NaN_3_ and 1% BSA) at 4 °C.

### Capture of spiked tumor cells in buffer and blood

Anti-EpCAM-MNs and anti-FRα-MNs of different concentrations were used to capture MCF7 and A2780 cells (1.0 × 10^5^ cells/mL in PBS), and the number of cells captured and uncaptured were all determined with a hemocytometer to calculate the corresponding capture efficiency. As controls, IMNs were used to treat Jurkat cells and A549 cells, while unmodified MNs were used to treat MCF7 and A2780 cells to investigate the specificity. Then, different incubation times (5, 10, 15, 20, and 30 min) were tested to select the optimal reaction time. Additionally, anti-EpCAM-MNs and anti-FRα-MNs were used to capture rare tumor cells in synthetic CTCs samples. Five groups of an extremely low concentration of Hoechst 33342-stained MCF7 and A2780 cells were added to whole blood with cell concentrations of approximately 5, 50, 100, 150, 200, and 300 cells/mL. A certain amount of the IMNs was added to the above samples for incubation at 37 °C. Then, they were isolated and washed with a magnetic scaffold. The captured and uncaptured cells were all counted to calculate the capture efficiency.

Then, anti-EpCAM-MNs and anti-FRα-MNs were used alone or in combination to capture the NSCLC cells (1.0 × 105 cells/mL in PBS); MCF7 and A2780 cells were the respective positive controls, and unmodified MNs were used to treat the cells to investigate the specificity. The numbers of captured and uncaptured cells were all determined with a hemocytometer to calculate the corresponding capture efficiency.

Additionally, anti-EpCAM-MNs and anti-FRα-MNs were used to capture rare tumor cells in synthetic CTCs samples. Hoechst 33342-stained SPC-A-1 and H157 cells were spiked into healthy human whole blood with a concentration of approximately 200 cells/mL to prepare closely mimicking clinical samples. In order to prove that the high enrichment efficiency was via the capture of EpCAM-positive cells and FRα-positive cells, we used a mixture of A2780 (FRα^+^/EpCAM^−/low^, 100 cells/mL) and MCF7 (EpCAM^+^/FR^−/low^, 100 cells/mL) cells as mimicking clinical samples. Then, they were isolated and washed with a magnetic scaffold. The captured and uncaptured cells were all counted to calculate the capture efficiency.

Additionally, anti-EpCAM-MNs and anti-FRα-MNs were combined to capture rare SPC-A-1 cells in mimicking clinical samples. Four groups of extremely low concentrations of Hoechst 33342-stained SPC-A-1 cells were added to whole blood with cell concentrations of approximately 5, 50, 100, 200, and 300 cells/mL. A certain amount of IMNs was added to the above samples for incubation at 37 °C. They were then isolated and washed with a magnetic scaffold. The captured and uncaptured cells were all counted to calculate the capture efficiency. Each group of spiked samples was tested in parallel for 4 times.

### Detection of NSCLC CTCs and CTC subtype analyses in peripheral blood samples

Our all experimental protocols were approved by the Ethics Committee of Renmin Hospital of Wuhan University. All experiments were performed in accordance with relevant guidelines and regulations.

Blood samples were drawn after gathering informed consent from 10 healthy donors and 41 NSCLC patients in Renmin Hospital of Wuhan University. Whole blood samples (4 mL) were collected in EDTA tubes (BD Biosciences, San Jose, CA, USA) and were used within 24 h. All of the blood was divided into 2 tubes (2 mL) and captured by anti-EpCAM-MNs or a combination of anti-EpCAM-MNs and anti-FRα-MNs. Blood samples were processed in a blind comparison study. Blood was incubated with IMNs for 15 min, and after magnetic separation, the captured cells were fixed with 4% paraformaldehyde (10 min), permeabilized with 0.1% Triton X-100 (10 min), blocked with 2% BSA (30 min), and incubated with Alexa Fluor^®^ 568-labeled anti-CK19 monoclonal antibody, Alexa Fluor^®^ 488-labeled anti-CD45 monoclonal antibody, and DAPI for 30 min. After thoroughly washing with PBS, the cells were coverslipped using antifade mounting medium. Fluorescence images were obtained by Olympus fluorescence microscope BX63 (Fig. 1(B)). The cells with CK19-positive and DAPI^-^positive but CD45-negative phenotypes were enumerated as CTCs.

For FRα-positive CTC detection, the blood samples from one NSCLC patient were processed with the whole immunomagnetic cell sorting process. After magnetic separation, the captured cells were fixed with 4% paraformaldehyde (10 min), permeabilized with 0.1% Triton X-100 (10 min), blocked with 2% BSA (30 min), and stained with anti-FRα monoclonal antibody for 30 min at 37 °C. After thoroughly washing with PBST (3 × 5 min), the cells were then incubated with Alexa Fluor^®^ 647 conjugated goat anti-mouse IgG (H + L) secondary antibody (Beyotime, Wuhan, China) for 30 min at 37 °C. After thoroughly washing with PBST, the cells were then incubated with Alexa Fluor^®^ 568-labeled anti-CK19 monoclonal antibody, Alexa Fluor^®^ 488-labeled anti-CD45 monoclonal antibody, and DAPI for 30 min. After thoroughly washing with PBS, the cells were coverslipped using antifade mounting medium. Fluorescence images were obtained by Olympus confocal Fluorescence microscope FV1200.

### Statistical analysis

Statistical analysis was performed with SPSS software, version 20.0 (SPSS Inc., Armonk, USA). The differences between two groups were evaluated with the two-tailed student’s *t* test. Statistical two-sided P values < 0.05 were considered to be statistically significant.

## Electronic supplementary material

Supplementary information
