# Supplementary material for: Combined use of EpCAM and FRα enables the high-efficiency capture of circulating tumor cells in non-small cell lung cancer
Source: Sci Rep. 2018 Jan 19;8:1188. doi: 10.1038/s41598-018-19391-1 (PMC5775318; doi:10.1038/s41598-018-19391-1)
Supplement: Supplementary file 1 — Supplementary information [file 41598_2018_19391_MOESM1_ESM.pdf]

Combined use of EpCAM and FRα enables the high-efficiency capture of circulating tumor cells in non-small cell lung cancer

Luojun Chen[1, +], Min Peng[1, +], Na Li[1, *], Qibin Song[1, *], Yi Yao[1], Bin Xu[1], Huali Liu[1], Peng Ruan[1]

[1] Department of Oncology, Renmin Hospital of Wuhan University, Wuhan,430060, China.

[*]corresponding: qibinsong@163.com; nalirenmin@163.com.

[+]these authors contributed equally to this work

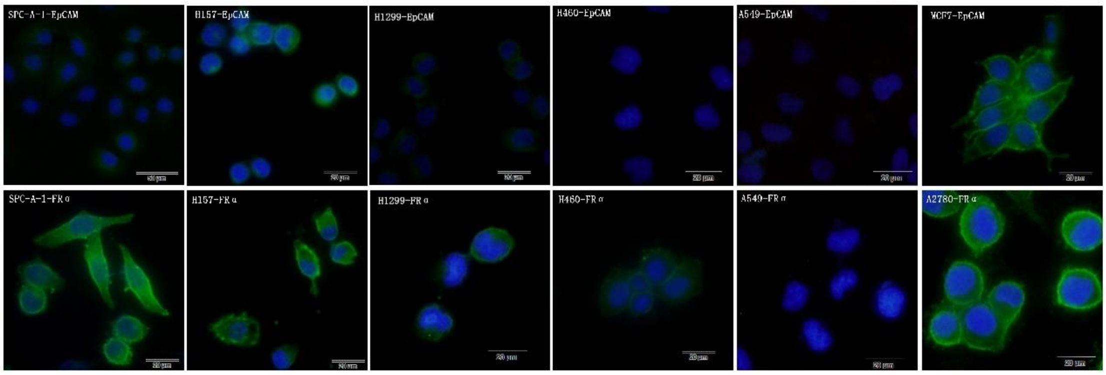

Fig.S1 Immunofluorescent detection of EpCAM and FRα expression in 5 types of NSCLC cells. EpCAM and FRα stained with Alexa Fluor® 488 are green at an excitation of 488 nm, and the nuclei stained with DAPI are blue at an excitation of 405 nm. As a positive control, we used EpCAM-stained MCF7 cells, which are highly expressed EpCAM, and FRα-stained A2780 cells, which are highly expressed FRα.

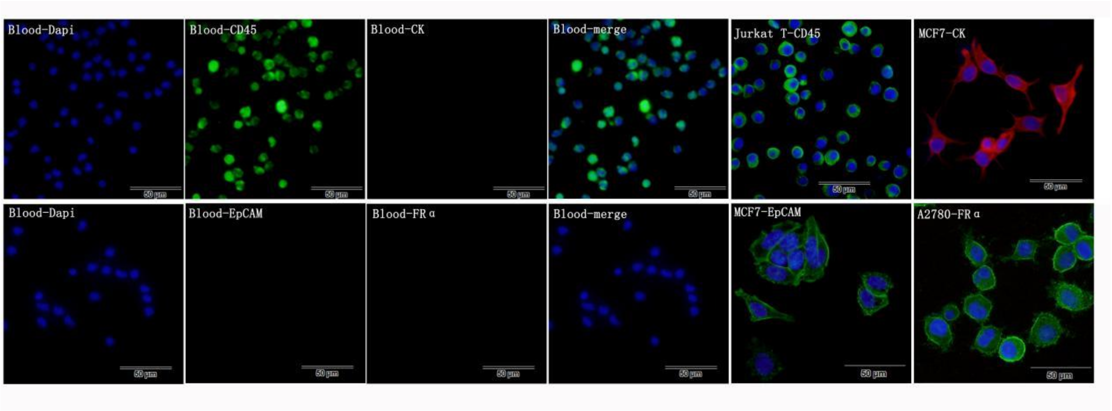

Fig. S2 Immunofluorescent detection of the expressions of CK19, CD45, EpCAM, and FRα in peripheral blood mononuclear cells: CD45s stained with Alexa Fluor® 488 are green at an excitation of 488 nm; CK19s stained with Alexa Fluor® 568 are red at an excitation of 568 nm; EpCAM and FRα stained with Alexa Fluor® 488 are green at an excitation of 488 nm; and the nuclei stained with DAPI are blue at an excitation of 405 nm. As a positive control, we used CD45-stained Jurkat cells, which highly express CD45, CK19-stained MCF7 cells, which highly express CK19, EpCAM-

stained MCF7 cells, which highly express EpCAM, and FRα-stained A2780 cells, which highly express FRα.

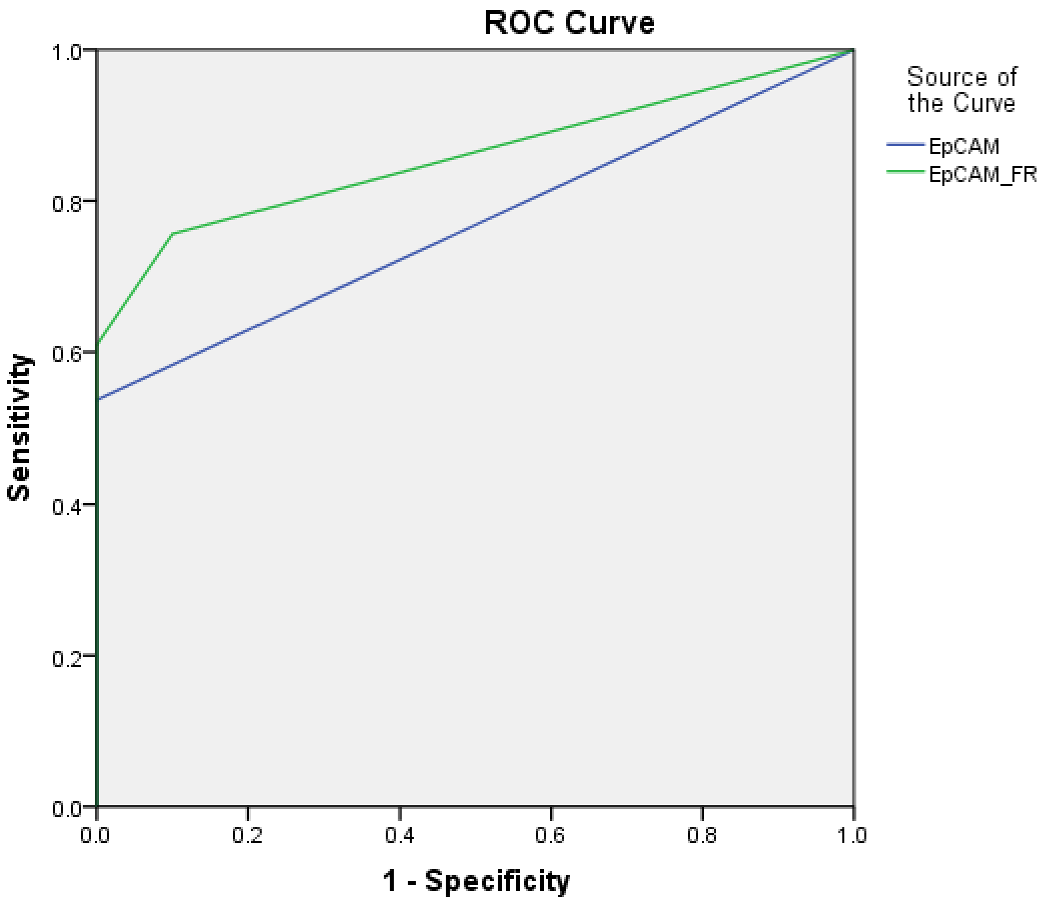

Fig. S3 ROC plots of using anti-EpCAM-MNs plus anti-FRα-MNs & anti-EpCAM-MNs to detect CTCs from 41 NSCLC patients and 10 healthy donors. The area under the receiver operating characteristic curve (AUC-ROC) analysis showed that the combination of anti-EpCAM-MNs and anti-FRα-MNs had a higher AUC (0.8585, 95% CI: 0.7579-0.9592, p=0.0005) than that of anti-EpCAM-MNs alone (0.7683, 95% CI: 0.6384-0.8982, p=0.0091).
